# Identification and Tetramer Structure of Hemin-Binding Protein SPD_0310 Linked to Iron Homeostasis and Virulence of Streptococcus pneumoniae

**DOI:** 10.1128/msystems.00221-22

**Published:** 2022-04-13

**Authors:** Kun Cao, Tianlong Zhang, Nan Li, Xiao-Yan Yang, Jianping Ding, Qing-Yu He, Xuesong Sun

**Affiliations:** a MOE Key Laboratory of Tumor Molecular Biology and Key Laboratory of Functional Protein Research of Guangdong Higher Education Institutes, Institute of Life and Health Engineering, College of Life Science and Technology, Jinan University, Guangzhou, China; b School of Medicine, Shanghai University, Shanghai, China; c Shanghai Institute of Biochemistry and Cell Biology, Center for Excellence in Molecular Cell Science, University of Chinese Academy of Sciences, Chinese Academy of Sciences, Shanghai, China; d Guangdong Provincial Key Laboratory of Medical Molecular Diagnostics, Institute of Aging Research, Guangdong Medical University, Dongguan, China; University of California San Diego

**Keywords:** tetramer structure, SPD_0310, *Streptococcus pneumoniae*, hemin binding, iron transportation, virulence

## Abstract

Iron and iron-containing compounds are essential for bacterial virulence and host infection. Hemin is an important supplement compound for bacterial survival in an iron-deficient environment. Despite strong interest in hemin metabolism, the detailed mechanism of hemin transportation in Gram-positive bacteria is yet to be reported. The results of our study revealed that the homologous proteins of SPD_0310 were significantly conservative in Gram-positive bacteria (*P < *0.001), and these proteins were identified as belonging to an uncharacterized protein family (UPF0371). The results of thermodynamic and kinetic studies have shown that SPD_0310 has a high hemin-binding affinity. Interestingly, we found that the crystal structure of SPD_0310 presented a homotetramer conformation, which is required for hemin binding. SPD_0310 can interact with many hemin-binding proteins (SPD_0090, SPD_1609, and GAPDH) located on the cell surface, which contributes to hemin transfer to the cytoplasm. It also has a high affinity with other iron transporters in the cytoplasm (SPD_0226 and SPD_0227), which facilitates iron redistribution in cells. More importantly, the knockout of the *spd_0310* gene (Δ*spd_0310*) resulted in a decrease in the iron content and protein expression levels of many bacterial adhesion factors. Moreover, the animal model showed that the Δ*spd_0310* strain has a lower virulence than the wild type. Based on the crystallographic and biochemical studies, we inferred that SPD_0310 is a hemin intermediate transporter which contributes to iron homeostasis and further affects the virulence of Streptococcus pneumoniae in the host. Our study provides not only an important theoretical basis for the in-depth elucidation of the hemin transport mechanism in bacteria but also an important candidate target for the development of novel antimicrobial agents based on metal transport systems.

**IMPORTANCE** Iron is an essential element for bacterial virulence and infection of the host. The detailed hemin metabolism in Gram-positive bacteria has rarely been studied. SPD_0310 belongs to the UPF0371 family of proteins, and results of homology analysis and evolutionary tree analysis suggested that it was widely distributed and highly conserved in Gram-positive bacteria. However, the function of the UPF0371 family remains unknown. We successfully determined the crystal structure of apo-SPD_0310, which is a homotetramer. We found that cytoplasmic protein SPD_0310 with a special tetramer structure has a strong hemin-binding ability and interacts with many iron transporters, which facilitates hemin transfer from the extracellular space to the cytoplasm. The results of detailed functional analyses indicated that SPD_0310 may function as a hemin transporter similar to hemoglobin in animals and contributes to bacterial iron homeostasis and virulence. This study provides a novel target for the development of antimicrobial drugs against pathogenic Gram-positive bacteria.

## INTRODUCTION

The trace element iron is essential for the survival and virulence of pathogenic bacteria. To maintain the balance of iron metabolism, bacteria generally acquire iron in two ways, competing with the host environment or degrading iron compounds, especially hemin (1). However, more than 99% of the iron in the human body is tightly bound by transferrin or hemoglobin (2–4). It has been reported that human hemoglobin is the target of many types of pathogenic hemin transporters, which is beneficial for bacteria to compete for hemin. For example, the iron-regulated surface determinant (IsdABC and IsdH) system removes hemin from hemoglobin and transfers it to other receptors of Staphylococcus aureus (5), and the haptoglobin-hemoglobin receptor (HpHbR) protein of Trypanosoma brucei *brucei* interacts with human haptoglobin and hemoglobin and then captures hemin (6).

Streptococcus pneumoniae (S. pneumoniae) is an important human pathogen that causes a variety of serious diseases, such as pneumonia, meningitis, and sepsis (7). Approximately 10.6 million invasive pneumococcal cases occur every year, of which nearly 2 million (especially children and the elderly) result in death from severe infection (8, 9). As with most bacteria, the pathogenicity of S. pneumoniae mainly depends on strong adhesion factors (for example, Ply, CbpA, PspA, and PcpA) to infect host cells (10, 11). Some studies have shown that iron or iron-containing compounds are the activators of virulence proteins and enzymes in S. pneumoniae (12).

In S. pneumoniae, three different iron uptake systems have evolved that promote effective absorption of iron sources. These are the pneumococcal iron ABC transporter (PitA) (13, 14), pneumococcal iron uptake (PiuA) (15, 16), and pneumococcal iron acquisition ATP-binding cassette (PiaA) (17), for Fe^3+^, hemin, and ferrichrome, respectively. GAPDH of S. pneumoniae is responsible for competing for hemin via interacting with human hemoglobin and plasminogen (6, 18). Moreover, the results of our previous study revealed a novel hemin transporter protein, SPD_1590 (19). However, the fate of hemin after entering the cytoplasm of S. pneumoniae remains unclear.

SPD_0310 is a cytosolic protein without a signal peptide and is defined as a hypothetical protein belonging to the UPF0371 family in the NCBI database. Several studies by our group have shown that this protein may participate in metal binding (14, 20, 21). First, iTRAQ-based proteomics have revealed that compared to wild-type S. pneumoniae D39 (WT-D39), the expression level of SPD_0310 protein increased approximately 3.5-fold in Δ*piaA* Δ*piuA* Δ*pitA* (triple mutant), in which three major iron transporters were simultaneously deleted (20, 22). Second, SPD_0310 interacts with the intracellular iron transporters SPD_0226 and SPD_0227 of S. pneumoniae (14). Third, the expression level of SPD_0310 in S. pneumoniae in manganese-deficient medium was 4.58-fold higher (*P < *0.05) than that in normal medium (21). Moreover, we demonstrated in a previous study that S. pneumoniae responds to manganese deficiency by developing a complete iron compensation mechanism (21). Based on these results, we speculated that SPD_0310 may function as an iron transporter or storage protein in S. pneumoniae. To uncover the biological function and contribution of SPD_0310 to bacterial virulence, we carried out a thorough investigation of the biochemical and structural characteristics *in vivo* and *in vitro*. The findings of this study will help in the effort to construct the complete iron metabolic pathway of S. pneumoniae and, consequently, aid in the development of new antibacterial drugs.

## RESULTS

### SPD_0310 belongs to the UPF0371 family, which is conserved in Gram-positive bacteria.

**FIG 1:**
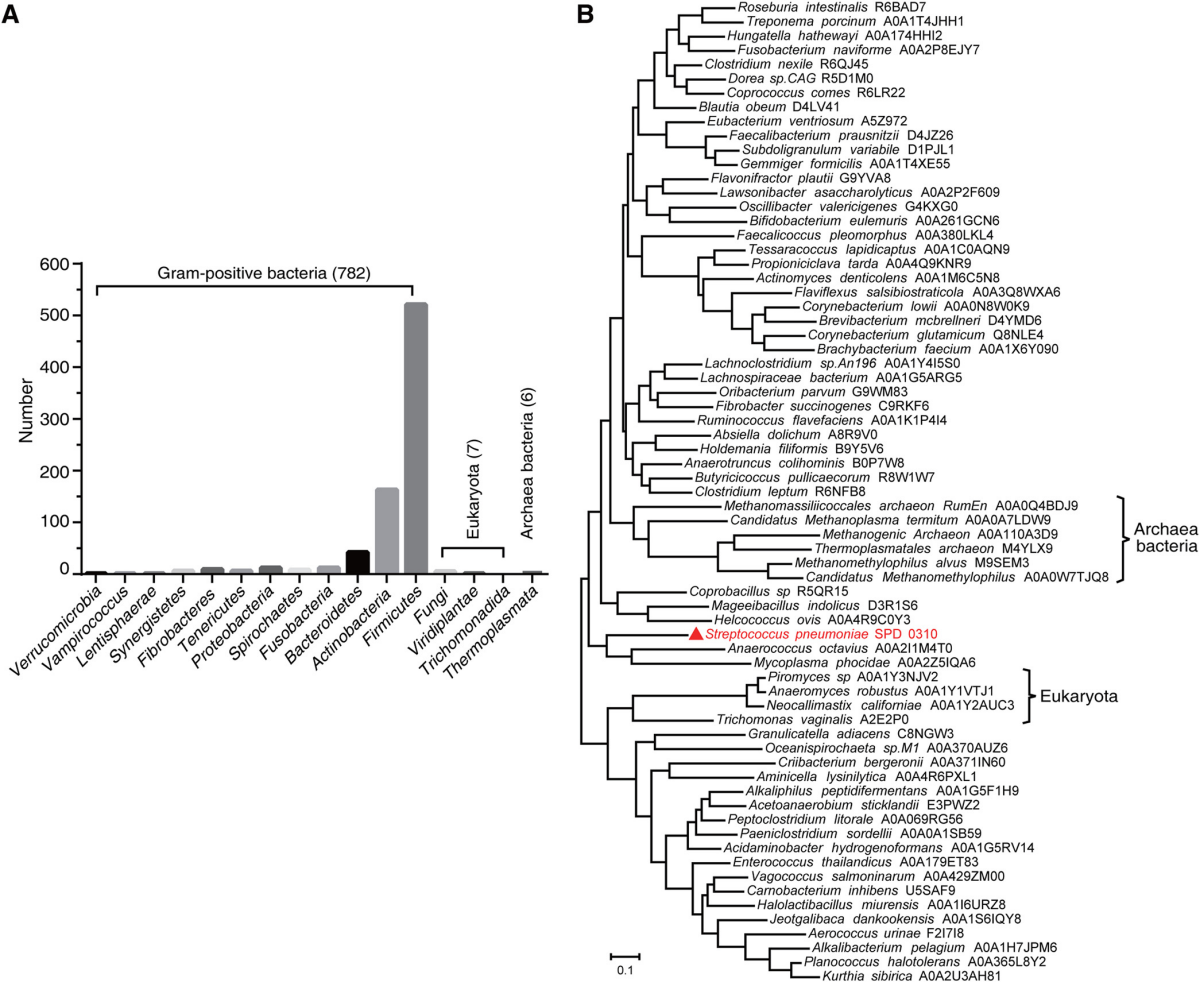

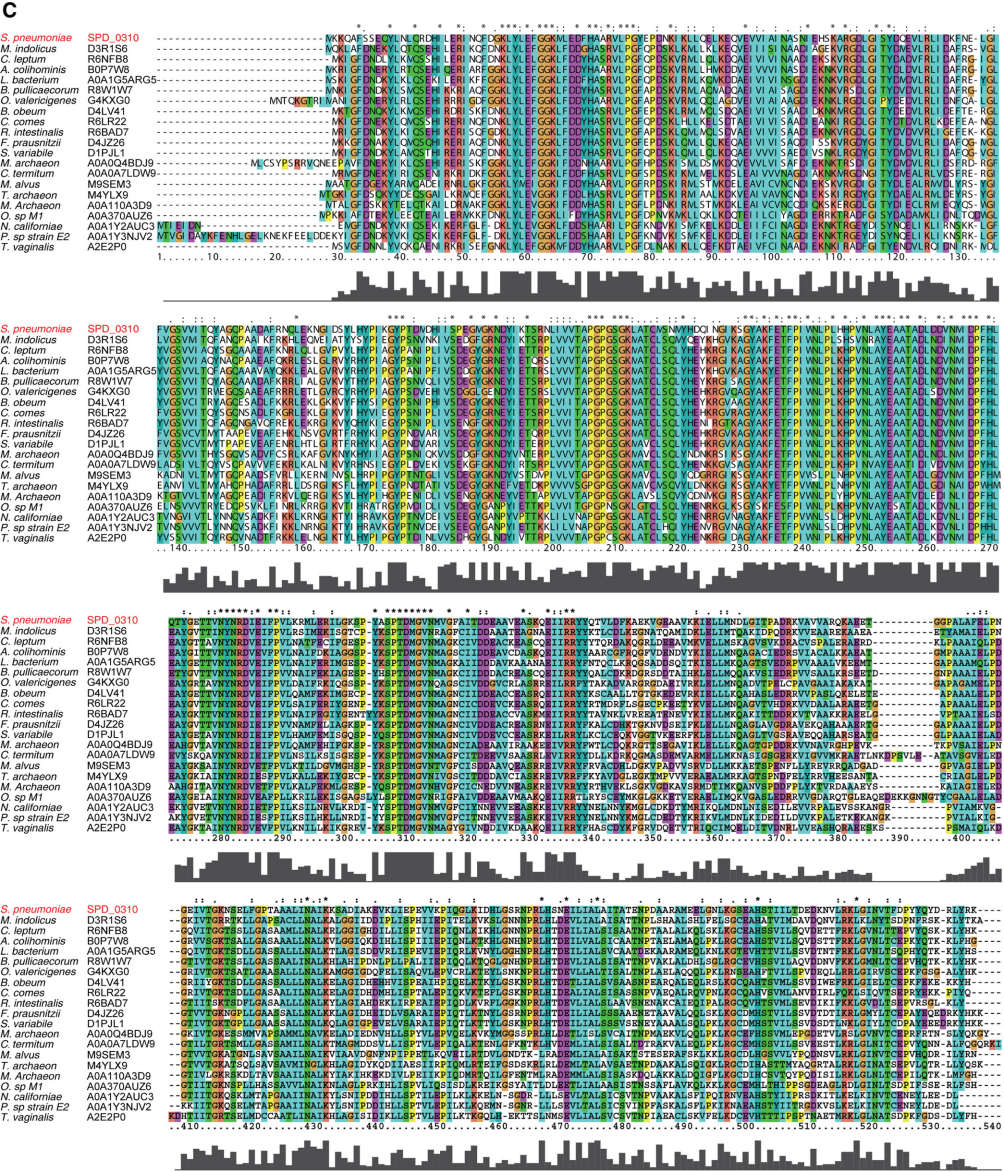
Evolutionary analysis of SPD_0310. (A) The bacteria containing the homologous protein of SPD_0310 distributed in different biological phyla. Distribution of homologous proteins of SPD_0310 in Gram-positive bacteria, archaeal bacteria, and eukaryota in the uniprotrefprot database using HMMER (>35% identity). (B) Molecular phylogenetic analysis of homologous proteins in representative species by the maximum likelihood method. (C) Multiple sequence alignment of SPD_0310 with homologous proteins of representative species.

To determine the conservation of SPD_0310 homologs in bacteria, we compared S. pneumoniae SPD_0310 with all completely sequenced microbiological genome sequences in the uniprotrefprot (v.2019_09) database, using the HMMER tool. In total, 795 proteins showed more than 35% sequence consistency with SPD_0310. Among these 795 homologous proteins, 782 proteins were significantly enriched in Gram-positive bacteria (*P < *0.001), whereas only 7 proteins occur in eukaryotes, such as Trichomonas vaginalis and Neocallimastix californiae, and 6 proteins were found in archaeal bacteria, such as Methanomethylophilus alvus and Thermoplasmatales archaeon (Fig. 1A). Moreover, the sequence identity of most proteins to SPD_0310 was more than 50%, suggesting that the SPD_0310 protein is highly conserved in Gram-positive bacteria. Interestingly, these homologous proteins were widely distributed in approximately 200 genera. Homologous proteins from 69 different species in representative genera were selected for phylogenetic analysis. The tree with the highest log likelihood (–34965.91) is shown in Fig. 1B. These proteins are separated by a short distance in the evolutionary tree, suggesting that UPF0371 family proteins may originate from the same ancestor in the evolutionary process. We selected 21 homologous proteins of microorganisms to perform sequence alignment analysis (Fig. 1C), which verified the high conservation of SPD_0310 protein in Gram-positive bacteria. However, according to the proportion of sequence similarity, the sequences of the UPF0371 family seemed to be divided into two segments, and the conservation of residues between 1 and 340 position was much higher than that of 341 to 540 position. So far, UPF0371 family proteins are still defined as hypothetical proteins.

### Crystal structure of the SPD_0310 homotetramer.

**FIG 2:**
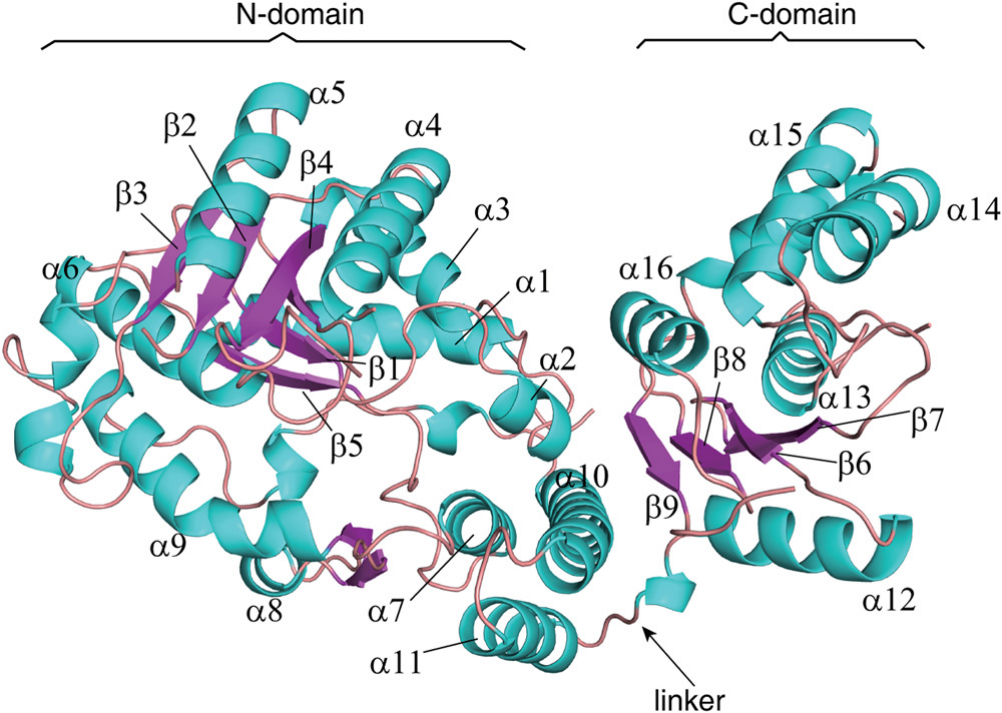
Detailed display of the structural characteristics of the SPD_0310 monomer.

To broaden the understanding of the biological function of UPF0371 family proteins, we purified apo-0310 (Fig. S1) and determined its crystal structure, which was solved using the molecular replacement method, and the refinement of the final atomic model converged to an *R*_work_ of 22.1% and an *R*_free_ of 26.5% to 2.7 Å resolution (PDB code 7F00). The data collection and refinement statistics for the structure are listed in Table 1. In the crystal structure of SPD_0310, there are two molecules in an asymmetric unit (AU) with a 2-fold noncrystallographic symmetry (NCS). Each monomer contains an N domain (residues 1 to 335) and a C domain (residues 343 to 485), connected by an unordered residue linker (residues 336 to 342). The N domain displays a mixed three-layer/architecture with five parallel β-strands (β3-β2-β4-β1-β5) sandwiched by five helices (α1 to α5) from one side and six helices (α6 to α11) from the other side. The C domain also assumes an α/β architecture and consists of four central β-strands (β7-β6-β8-β9) that are parallel, with the exception of β6. Helix α12 lies against the β-strands of the C domain from one side and helices α13–α16 from the other side (Fig. 2). These results showed that only the N domain of SPD_0310 has a hydrophobic pocket, while the C domain does not.

**TABLE 1:** Summary of diffraction data and structure refinement statistics

Measurement	
Diffraction data	Value
Wavelength (Å)	0.9777
Space group	*P*4_1_2_1_2
Cell parameters	
*a*, *b*, *c* (Å)	87.85, 87.85, 326.12
Resolution (Å)	50.0–2.70 (2.80–2.70)^*a*^
Observed reflections	154,871
Unique reflections (I/σ[I] > 0)	36,236
Avg redundancy	4.3 (4.2)
Avg I/σ(I)	30.9 (2.8)
Completeness (%)	99.7 (99.9)
*R*_merge_ (%)	4.5 (55.2)
Refinement and structure model	
Reflections (*Fo ≥ 0σ*[*Fo*])	
Working set	31,985
Test set	1,795
*R*_work_/*R*_free_ (%)	22.1 /26.5
No. of protein atoms	7,322
No. of solvent atoms	35
Avg B factor (Å^2^)	
All atoms	53.7
Protein atoms	53.8
Solvent atoms	43.4
RMS deviation	
Bond lengths (Å)	0.007
Bond angles (°)	1.12
Ramachandran plot (%)^*b*^	
Most favored regions	97.2
Other allowed regions	2.8
Outliers	0.0

a Numbers in parentheses represent the highest resolution shell.

b Statistics of the Ramachandran plot were analyzed using MolProbity.

10.1128/msystems.00221-22.1 FIG S1 Protein expression and Identification by mass spectrometry. Download FIG S1, EPS file, 2.5 MB. Copyright © 2022 Cao et al. 2022 Cao et al. https://creativecommons.org/licenses/by/4.0/ This content is distributed under the terms of the Creative Commons Attribution 4.0 International license.

**FIG 3:**
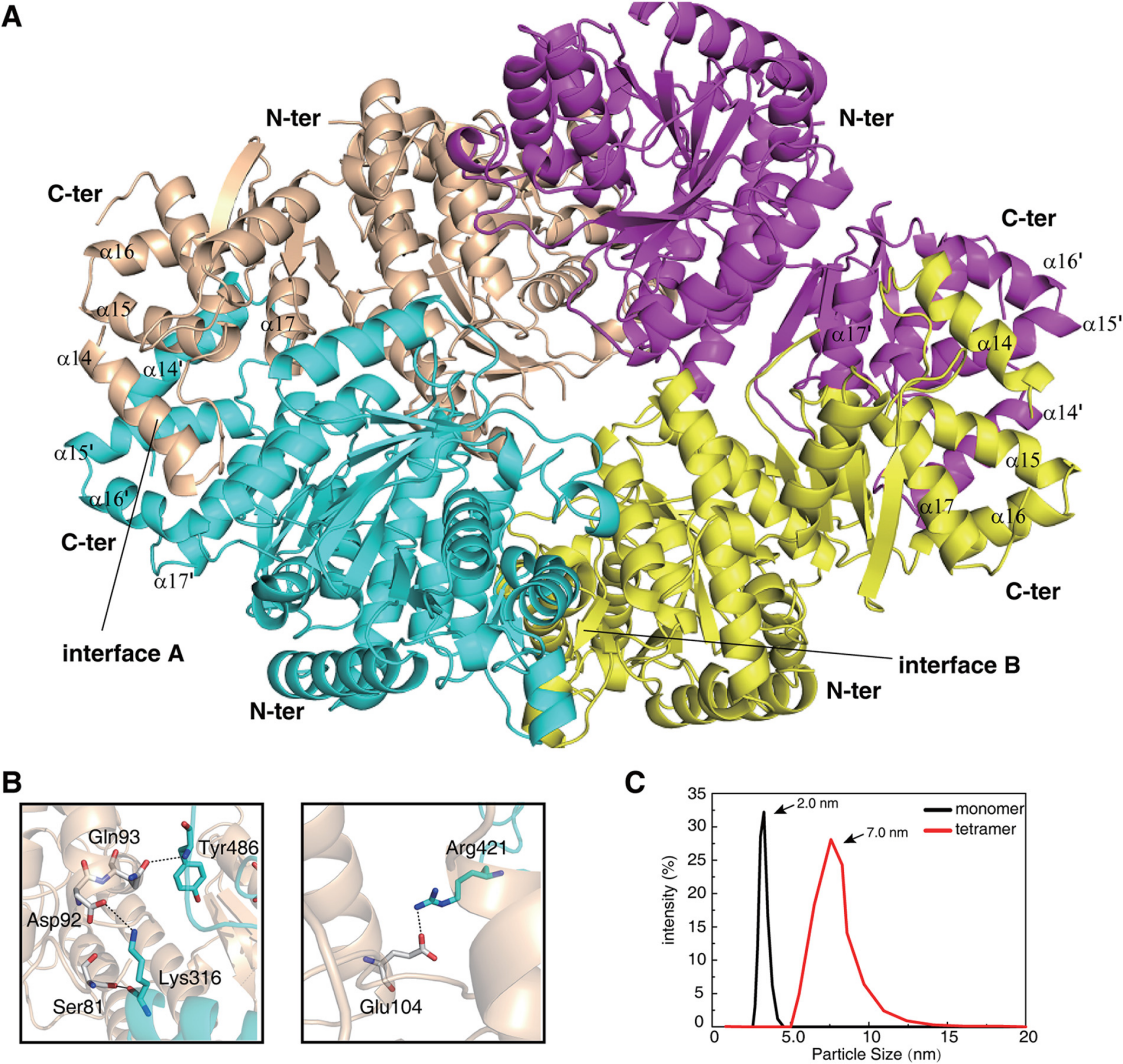
Overall structure of the SPD_0310 homotetramer protein. (A) Crystal structure of the tetramer of apo-0310 and the display of its main interaction region. (B) H-bonds between the N terminus and C terminus in monomer molecules. (C) Detection of particle size of monomer or tetramer of SPD_0310 in solution by DLS.

The entire SPD_0310 homotetramer was arranged with a crystallographic 2-fold symmetry of each of the two AUs (Fig. 3A). The tetrameric interface was predicted with PISA (23), leading to a stable homotetramer formation of SPD_0310 in solution with a ΔG of dissociation (ΔGdiss) of 18.4 kcal/mol and a buried surface area (BSA) of 11704.6 Å^2^ (Table 2). The interaction within the tetramer protein was mainly concentrated in three regions—N to N domain, C to C domain, and N to C domain. Here, the H-bond interactions between the N and C domains are shown as Gln93-Tyr486, Asp92-Lys316, Ser81-Lys316, and Glu104-Arg421 (Fig. 3B). To confirm the oligomeric state of SPD_0310, both the sizes of protein particles in the natural state and in the monomeric state obtained by adding SDS were detected by dynamic light scattering (DLS) (Fig. 3C). SPD_0310 protein showed good solution homogeneity in the natural state, with an equivalent sphere particle size of approximately 7.0 nm, whereas the size of the corresponding monomer was only 2 nm (24). These results indicate that SPD_0310 exists in a tetrameric state in solution, which is in agreement with the crystal structure. These crystallographic data of the tetramer enrich the basic understanding of the UPF0371 family protein from the perspective of structural biology.

**TABLE 2:** Analysis of protein interfaces

Size	ASA (Å^2^)	BSA (Å^2^)	ΔGdiss (kcal/mol)
Homotetramer	78,068.0	11,704.6	18.4
Homodimer-A	41,297.2	3,589.4	10.1
Homodimer-B	42,769.6	2,117.0	0.4

### H-bonds and hydrophobic interactions stabilize the tetramers.

**FIG 4:**
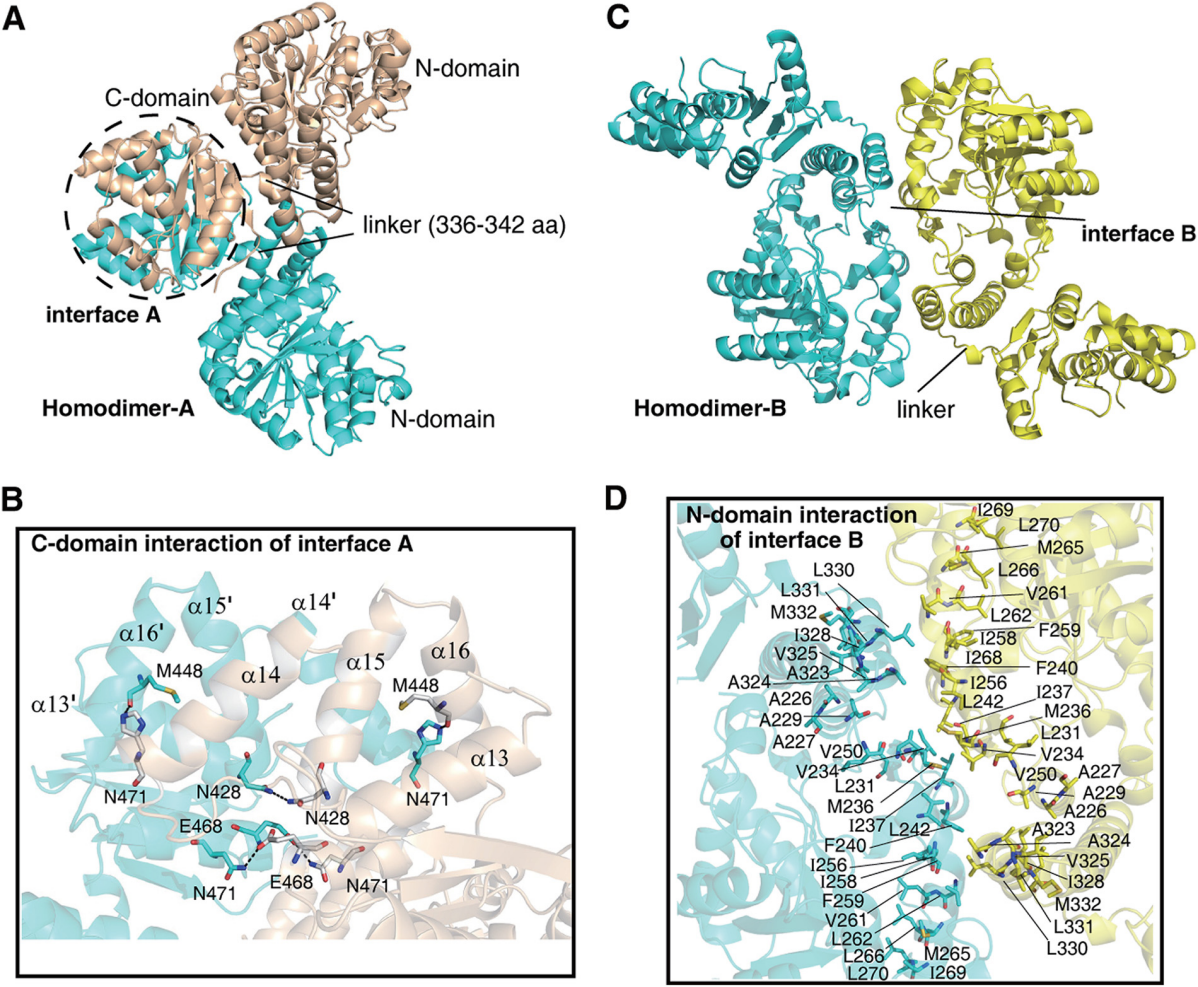
The interaction of two homodimers in the tetramer SPD_0310. (A) Interface A of homodimer A shown through the interaction of the C domain (black dashed line). (B) Interactions at the C-domain homodimer interface for detail. H-bonds are indicated with dashed lines. (C) Interface B of homodimer B relying on local hydrophobic interaction to facilitate conformational stability. (D) The hydrophobic residues of N domains in two monomers labeled by line.

The tetramers of SPD_0310 were formed through the interaction of two types of homodimers with cross-linking, homodimers A and B. The majority of the contacts come from homodimer A (Fig. 4A), accessible surface area (ASA) of 41297.2 Å^2^ and BSA of 3589.4 Å^2^, which needed a ΔGdiss as high as 10.1 kcal/mol (Table 2). In contrast, the homodimer B, with an ASA of 42769.6 Å^2^ and a BSA of 2117.0 Å^2^, showed a ΔGdiss of 0.4 kcal/mol (Table 2). The tetramer was tightly connected by four important contact surfaces (symmetry) between each monomer and interfaces A and B. Interface A consists of two C domains, indicating that it depends on the residues to form intermolecular forces, which further indicates a large area of hydrophobic contact between α13/α14/α15/α16 and α13′/α14′/α15′/α16′, and some representative hydrogen bonds are formed by Asn471 to Met488, Asn428 to Asn428, and Glu468 to Asn471 (Fig. 4B). The results showed that interface B consists of two N domains, which mainly rely on hydrophobic forces to maintain the connection (Fig. 4C). Each monomer contributed to the formation of a hydrophobic groove with as many as 26 hydrophobic residues on the interface (Fig. 4D). Therefore, these results suggested that the stable conformation of SPD_0310 can exist naturally as a tetramer. We also tried to screen the complex crystals of SPD_0310 and hemin through cocrystallization and collected diffraction data of crystals obtained under different conditions with low salt but failed to obtain the structure of the complex.

Among all the homologous proteins of SPD_0310, only the three-dimensional structure of DIP2346 protein (PDB code 3BH1) of Corynebacterium diphtheriae was displayed in the PDB database, but its function has not been reported so far. The independent sequence comparison showed that SPD_0310 and 3BH1 share 54% homology. The structural similarities of the two proteins were as follows: an apo-form tetramer with a double domain, a hydrophobic active pocket at the N domain, and the connection method between monomers. The similarity of the N terminals of the two proteins is 58% similarity, while that of the C terminals was 43%. The major difference between the two proteins in the structure is that SPD_0310 possesses a larger tetramer central channel than that in 3BH1.

### Thermodynamic and kinetic characterization of specific binding between hemin and protein.

**FIG 5:**
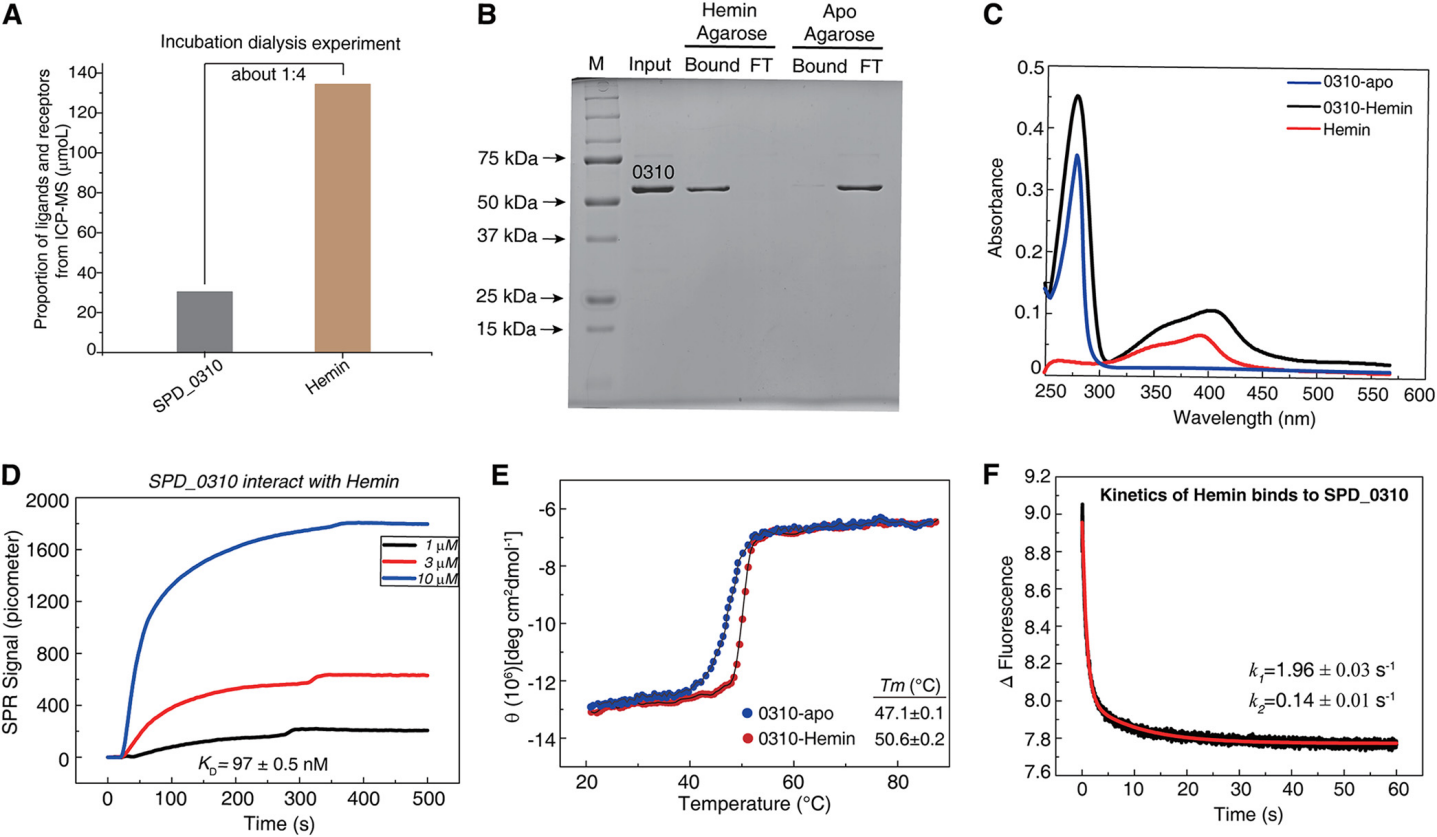
Biochemical characteristics of thermodynamics and kinetics of SPD_0310 protein binding to hemin. (A) Determination of the binding between SPD_0310 and hemin by dialysis combined with ICP-MS. The SPD_0310 was incubated with excess hemin for 6 h and then dialyzed three times and concentrated with a centrifugal filter unit. The SPD_0310 concentration was measured with a bicinchoninic acid (BCA) kit, and the content of iron was detected by ICP-MS. (B) Detection of the binding of SPD_0310 with hemin by hemin-agarose and SDS-PAGE gel. The input was SPD_0310 protein which was incubated with hemin-agarose or apo-agarose and slightly centrifuged. Then the supernatant (hemin agarose FT or apo-agarose FT) and precipitate (hemin agarose bound or apo-agarose bound) were detected by SDS-PAGE. (C) UV-Vis absorption spectra of hemin-0310. The specific absorption peaks of hemin, apo SPD_0310, and hemin-bound were shown at 400 nm (red line), 280 nm (blue line), and 410 nm (black line), respectively. (D) The binding of the protein to hemin detected by SPR. (E) The thermal stability of the proteins detected by circular dichroism (CD). The spectrum values were collected at 208 nm from 20°C to 90°C. (F) Detection of the binding kinetics between apo-0310 and hemin by the stopped-flow method.

In our previous research, the quantitative proteomics and glutathione-S-transferase (GST) pulldown data suggested that SPD_0310 may be involved in iron metabolism of S. pneumoniae and have the ability to bind iron or iron-containing compounds (20–22). Moreover, the UPF0371 family proteins have abundant histidine and aspartic and glutamic acids (HDE), implying strong metal-chelating properties (25). Dialysis combined with inductively coupled plasma mass spectrometry (ICP-MS) was used to detect the binding of SPD_0310 with different ligands, such as Fe^3+^, hemin, and ferrichrome. The results showed that SPD_0310 only bound hemin, not Fe^3+^, and ferrichrome. The residual protein and iron contents were 30 μM and 134 μM, respectively, indicating that hemin is the binding substrate of SPD_0310, and the binding ratio of the tetramer protein to hemin was approximately 1:4 (Fig. 5A). Next, a hemin-agarose assay was used to validate the hemin-binding ability of SPD_0310. Compared with the apo-agarose group (negative control), there was SPD_0310 protein in the precipitate of the hemin-agarose group, but not in the supernatant (Fig. 5B), indicating a hemin-binding ability of SPD_0310. Further, UV-visible (UV-Vis) spectra showed that the specific absorption peak of hemin itself was 400 nm and that of apo SPD_0310 protein was 280 nm. However, the hemin-bound SPD_0310 complex not only showed the absorption peak of protein at 280 nm, but also showed a new absorption peak at 410 nm (Fig. 5C). The shift of the specific absorption peak from 400 nm to 410 nm is due to the binding of hemin to the protein (26). These results demonstrated that the specific substrate of SPD_0310 is hemin.

We further determined the binding affinity of the 0310 protein with hemin by surface plasmon resonance (SPR). The SPR result showed that the dissociation constant of SPD_0310 for hemin was 9.69 ± 0.50 × 10^−8^ M (Fig. 5D), indicating that SPD_0310 has a strong binding ability for hemin. Thermal stability analysis showed that the midpoint temperature (*T_m_*) values of apo- and hemin-0310 were 47.1°C ± 0.1°C and 50.6°C ± 0.2°C (Fig. 5E), respectively. The conformational change caused by hemin binding may result in a tight and stable structure, leading to enhanced resistance of SPD_0310 to thermal denaturation.

Subsequently, a stopped flow assay based on fluorescence mode was used to detect the hemin-binding kinetics of the protein (Fig. 5F). The binding is a very fast kinetics with a two-component reaction (*k*_1_ = 1.96 ± 0.03 s^−1^ and *k*_2_ = 0.14 ± 0.01 s^−1^, A_1_ = 0.784 ± 0.005 and A_2_ = 0.299 ± 0.002). The binding process was composed of a fast reaction for 0.5 s and a slow reaction for approximately 7.0 s.

### SPD_0310 contributes to iron homeostasis in bacteria.

To further investigate the biological function of the tetramer protein SPD_0310 *in vivo*, we constructed a gene knockout strain (Δ*spd_0310*) and a complementary strain (*spd_0310* complement) by homologous recombination. Some studies have shown that pIB169 (p169), a rolling-circle replicon (RCR) plasmid, was more suitable for the cloning and gene expression in Streptococcus, because its *veg* promoter (P_veg_) has high homology with Gram-positive bacteria, and its structural composition is very simple (polyclonal sites, chloramphenicol resistance, and P_veg_), and thus it does not increase a lot of fitness costs (27). Thus, p169 was used to construct the *spd_0310* complement strain in this study. All mutant strains were confirmed by gene sequencing and Western blotting (Fig. S2A). The pneumococcal surface antigen A (PsaA) was used as the internal reference in Western blotting because its expression was not affected by *spd_0310* gene knockout, and the antibody against PsaA had been prepared in our previous study (28).

10.1128/msystems.00221-22.2 FIG S2 Verification of *spd_0310* gene knockout and complement, detection of fitness cost of p169 empty plasmid and SPD_0310 interacting protein. Download FIG S2, EPS file, 1.9 MB. Copyright © 2022 Cao et al. 2022 Cao et al. https://creativecommons.org/licenses/by/4.0/ This content is distributed under the terms of the Creative Commons Attribution 4.0 International license.

**FIG 6:**
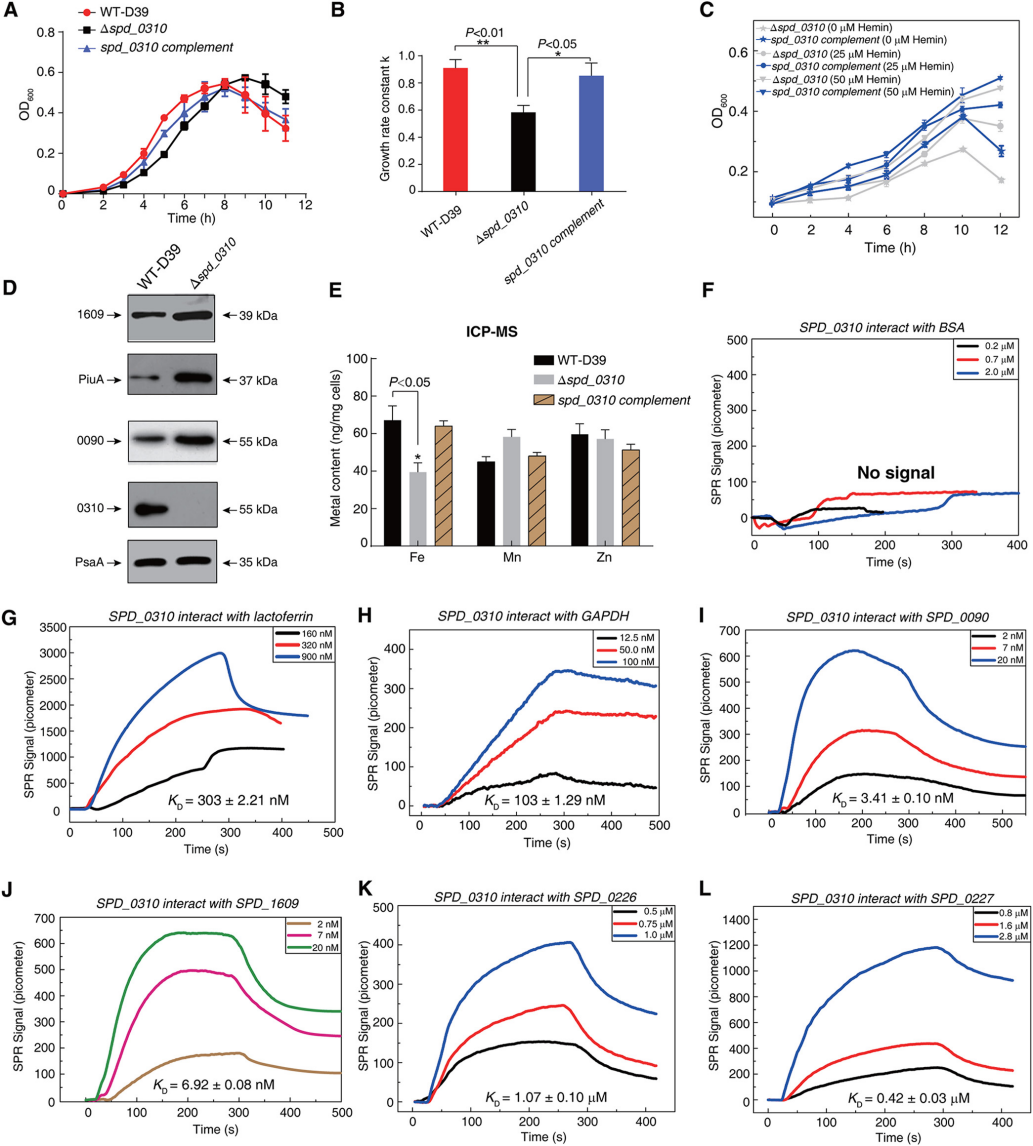
Detection of bacterial iron content and expression of crucial iron transporters. (A) Growth curves of WT-D39, Δ*spd_0310*, and *spd_0310* complement strains cultured in Todd-Hewitt broth with 0.5% yeast extract (THYE) medium. (B) The growth rate in the logarithmic growth period corresponding to the bacterial growth curve. (C) Growth curves of Δ*spd_0310* and *spd_0310* complement strains in the presence of 25 and 50 μM hemin in C+Y culture medium. (D) The content of metal ions in bacteria. WT-D39, Δ*spd_0310*, and *spd_0310* complement strains were cultured in THYE medium and collected under the same conditions. After removal of the residual metal ions from the culture medium, the metal ion content in bacteria was detected by ICP-MS. (E) Expression of several typical iron transporters in the Δ*spd_0310* strain detected using Western blotting. (F to L) The interaction between apo-0310 (target protein) and known iron transporters verified by SPR; BSA protein (F) and lactoferrin (G) were used as the negative control and positive control, respectively. The concentrations of candidate protein samples were used as follows: 12.5, 50, and 100 nM GAPDH (H); 2.0, 7.0, and 20 nM SPD_0090 (I); 2.0, 7.0, and 20 nM SPD_1609 (J); 0.5, 0.75, and 1.0 mM SPD_0226 (K); and 0.8, 1.6, and 2.8 mM SPD_0227 (L). The results in panels B and E were displayed as means ± standard deviation (SD), and the significance of differences was determined by two-tailed Student’s *t* test (*, *P < *0.05; **, *P < *0.01).

Growth curves were determined to investigate the effect of deletion of *spd_0310* on bacterial survival. The results showed that the logarithmic state of Δ*spd_0310* was retarded, whereas a better recovery of growth was observed in the complement strain (Fig. 6A). We calculated the growth rate of WT-D39, the Δ*spd_0310* mutant, and a complement strain in the logarithmic growth period. Compared with the WT-D39, the growth rate of the Δ*spd_0310* mutant strain decreased significantly (*P < *0.01) (Fig. 6B), and the growth rate of the complement strain was significantly faster than that of the Δ*spd_0310* mutant (*P < *0.05) and similar to that of WT-D39. Although we have avoided introducing external factors to bacteria as much as possible, the introduction of *erm* into Δ*spd_0310* and the p169 plasmid into the Δ*spd_0310* complement might cause a fitness cost. Thus, after screening the positive clone, the mutant strain was passaged 5 times in Todd-Hewitt broth with 0.5% yeast extract (THYE) medium without erythromycin. In order to test whether the introduction of plasmid causes the adaptive cost of bacteria, we also measured the growth curves of the Δ*spd_0310* mutant containing the p169 empty plasmid. The result indicated that p169 only had a little effect in the logarithmic growth period (*P > *0.05) (Fig. S2B).

Next, to find out the correlation between SPD_0310 protein and hemin utility, we determined the growth curves of Δ*spd_0310* and the *spd_0310* complement strain in the Casein-based semisynthetic liquid culture (C+Y) restricted medium (29) supplemented with different concentrations of hemin (Fig. 6C). The results showed that the growth state of the Δ*spd_0310* complement strain was better than that of the Δ*spd_0310* mutant, which was positively correlated with the concentration of hemin. Moreover, the expression levels of representative iron transporters PiuA, SPD_1609, and SPD_0090 were simultaneously upregulated in Δ*spd_0310* compared with WT-D39 (Fig. 6D), which suggests iron deficiency in the knockout strain. To verify whether iron metabolism was affected, the metal contents of the WT-D39, mutant, and complement strains were detected using ICP-MS. The iron content in Δ*spd_0310* was significantly lower than that of WT-D39 (*P = *0.039), whereas the contents of manganese and zinc were not affected (*P > *0.05) (Fig. 6E). The level of iron in the *spd_0310* complement strain was recovered and found to be close to that of WT-D39. These results suggested that deletion of *spd_0310* decreased the intracellular iron levels in S. pneumoniae. Therefore, we speculated that *spd_0310* was involved in iron metabolism of the bacterium.

To further investigate the detailed function of SPD_0310 in iron metabolism of S. pneumoniae, GST pulldown combined with MS was performed to identify the potential proteins interacting with SPD_0310 (Fig. S2C). To find more potential proteins, the proteins in the whole strip lanes were digested with trypsin and identified with MS. Compared with these control groups, the additional proteins in the experimental group were considered the candidate interacting proteins. In total, 28 candidate proteins were identified, and the corresponding scores derived by the Mascot system according to the coverage of identified peptides are listed in Table 3. These proteins are mainly ABC transporters or involved in energy metabolism. Among these proteins, the lipoproteins SPD_0090, SPD_1609, and GAPDH were expressed, purified, and initially characterized as iron transporters in our previous studies (14, 30). To accurately verify the interaction of these candidate proteins with SPD_0310, SPR was performed to detect binding affinity, and bovine serum albumin (BSA protein) and lactoferrin were used as the negative control and positive control, respectively. The reaction signal was not detected in the interaction between BSA protein and SPD_0310 (Fig. 6F), but there was a strong signal in the reaction between lactoferrin and SPD_0310 with dissociation constant (*K_D_*) values of 303 ± 2.21 nM (Fig. 6G). These three lipoproteins showed a strong affinity for SPD_0310, showed as follows: GAPDH, SPD_0090, and SPD_1609 had *K_D_* values of 103 ± 1.29 nM (Fig. 6H), 3.41 ± 0.01 nM (Fig. 6I), and 6.92 ± 0.08 nM (Fig. 6J), respectively. In addition, our previous research indicated that SPD_0310 was pulled down by SPD_0226-GST and SPD_0227-GST of S. pneumoniae D39 (14), respectively. In this study, the interaction intensities between SPD_ 0310 and SPD_0226 or SPD_0227 were also measured by SPR; the resulting dissociation constants (*K_D_*) were 1.07 ± 0.10 μM (Fig. 6K) and 0.42 ± 0.03 μM (Fig. 6L), respectively. SPD_0310 has strong affinity with these iron transporters anchored on the membrane, which makes it possible to transfer hemin from the extracellular space to the cytoplasm.

**TABLE 3:** The candidate proteins interacting with SPD_0310 identified in GST pulldown

Reference no.	Protein name	Description	Score
gi|116515427	SPD_1609	ABC transporter, substrate-binding	623
gi|116516928	SPD_0090	ABC transporter, substrate-binding	127
gi|116516065	SPD_1621	ABC transporter, ATP-binding/permease	36
gi|116515752	SPD_1297	Pyridoxine biosynthesis protein	88
gi|116516240	SPD_1487	Phospho-binding transcriptional regulator	33
gi|116516896	SPD_0913	Hypothetical protein	114
gi|116516442	Gap	Gly-3-pho dehydrogenase (GAPDH)	733
gi|116516995	GpsA	NAD(P)H-dependent glycerol-3-phosphate dehydrogenase	47
gi|116515997	PfkA	ATP-dependent 6-phosphofructokinase	531
gi|116516051	Cps2L	Glucose-1-phosphate thymidylyltransferase	39
gi|116515565	LacR1	Lactose phosphotransferase repressor	35
gi|116515434	SPD_0205	50S ribosomal protein L5	21
gi|116516989	SPD_0948	ATP-grasp domain-containing protein	15
gi|116515333	SPD_0921	Site-specific recombinase, resolvase family protein	13
gi|116516380	SPD_0242	Uncharacterized protein	16
gi|116515705	RpsH	30S ribosomal protein S8	20
gi|116517120	SPD_0603	Peptidase, M50 family protein	16
gi|116515631	ParC	DNA topoisomerase IV subunit A	15
gi|116516619	SPD_0378	Enoyl-CoA hydratase	14
gi|116517148	SPD_1021	Voltage-gated chloride channel family protein	18
gi|116516069	SPD_2022	ATP-dependent Clp protease, ATP-binding subunit	13
gi|116516062	PurH	Phosphoribosylaminoimidazolecarboxamide formyltransferase/IMP cyclohydrolase	17
gi|116516037	SPD_0554	ABC transporter, ATP-binding protein	15
gi|116515736	MurB	UDP-*N*-acetylenolpyruvoylglucosamine reductase	16
gi|116516609	PrsA	Ribose-phosphate pyrophosphokinase	19
gi|116516167	SPD_1736	Uncharacterized protein	16
gi|116515771	DppC	Oligopeptide ABC transporter, permease protein	15
gi|116517172	MalX	Maltose/maltodextrin ABC transporter, maltose/maltodextrin-binding protein	16

### SPD_0310 enhances the pathogenicity of S. pneumoniae in host cells.

**FIG 7:**
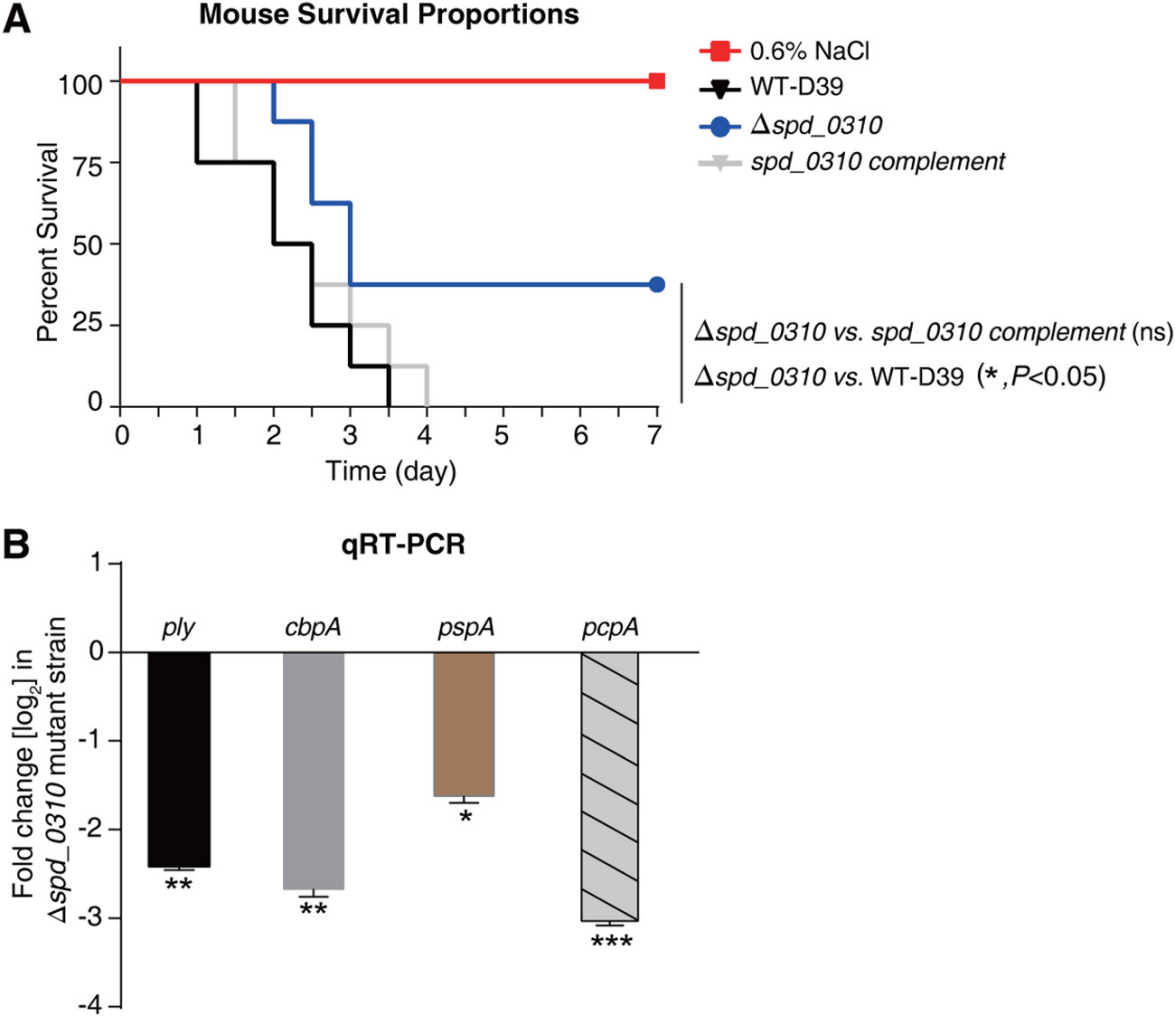
Animal experiments. (A) Survival curves of mice infected with 2 × 10^6^ CFU of WT-D39, Δ*spd_0310* mutant, or *spd_0310* complement strains by intravenous injection through the tail vein; *n* = 8 animals per group. The survival proportion was analyzed using the log-rank (Mantel-Cox) test between the Δ*spd_0310* mutant and WT-D39 strains. (B) qRT-PCR expression analysis of *ply*, *cbpA*, *pspA*, and *pcpA* in the WT-D39, Δ*spd_0310* mutant, and *spd_0310* complement strains. The relative gene expression was calculated with 16s rRNA as the reference gene. The results were displayed as means ± SD, and the significance of differences was determined by two-tailed Student’s *t* test (*, *P < *0.05; **, *P < *0.01; ***, *P < *0.001).

To reflect the effect of SPD_0310 on bacterial pathogenicity, BALB/c mice were used as the infected specimens. Each group contained eight mice that were infected with WT-D39, Δ*spd_0310*, and *spd_0310* complement strains through the tail vein (31), respectively. As shown in Fig. 7A, the survival rate of the Δ*spd_0310*-infected mice was significantly higher than that of WT-D39-infected mice (*P < *0.05), whereas the rate of *spd_0310* complement-infected mice was partially recovered. The reason for this incomplete recovery may be that the complement strain needs to deal with the adaptive cost of multicopy plasmid or the partial loss of plasmid caused by the absence of chloramphenicol pressure in mice. To further investigate the reason of the increase of survival rate of infected mice caused by the *spd_0310* gene knockout strain, the mRNA levels of four crucial virulence factors were detected by real-time quantitative PCR (qRT-PCR) in WT-D39 and Δ*spd_0310*. 16S rRNA was the internal reference of gene expression because there was no difference in its expression level between the experimental group and the control group. The quantitative results indicated that the genes *ply*, *cbpA*, *pspA*, and *pcpA* were downregulated 5.2-fold, 6.7-fold, 3.6-fold, and 8.1-fold, respectively, in the Δ*spd_0310* mutant strain compared with the WT-D39 parent strain (Fig. 7B). These results suggested that the tetramer protein SPD_0310 is very important for bacterial survival and virulence in the host.

### Hemin binding stabilizes the overall structure of double-domain SPD_0310 proteins.

**FIG 8:**
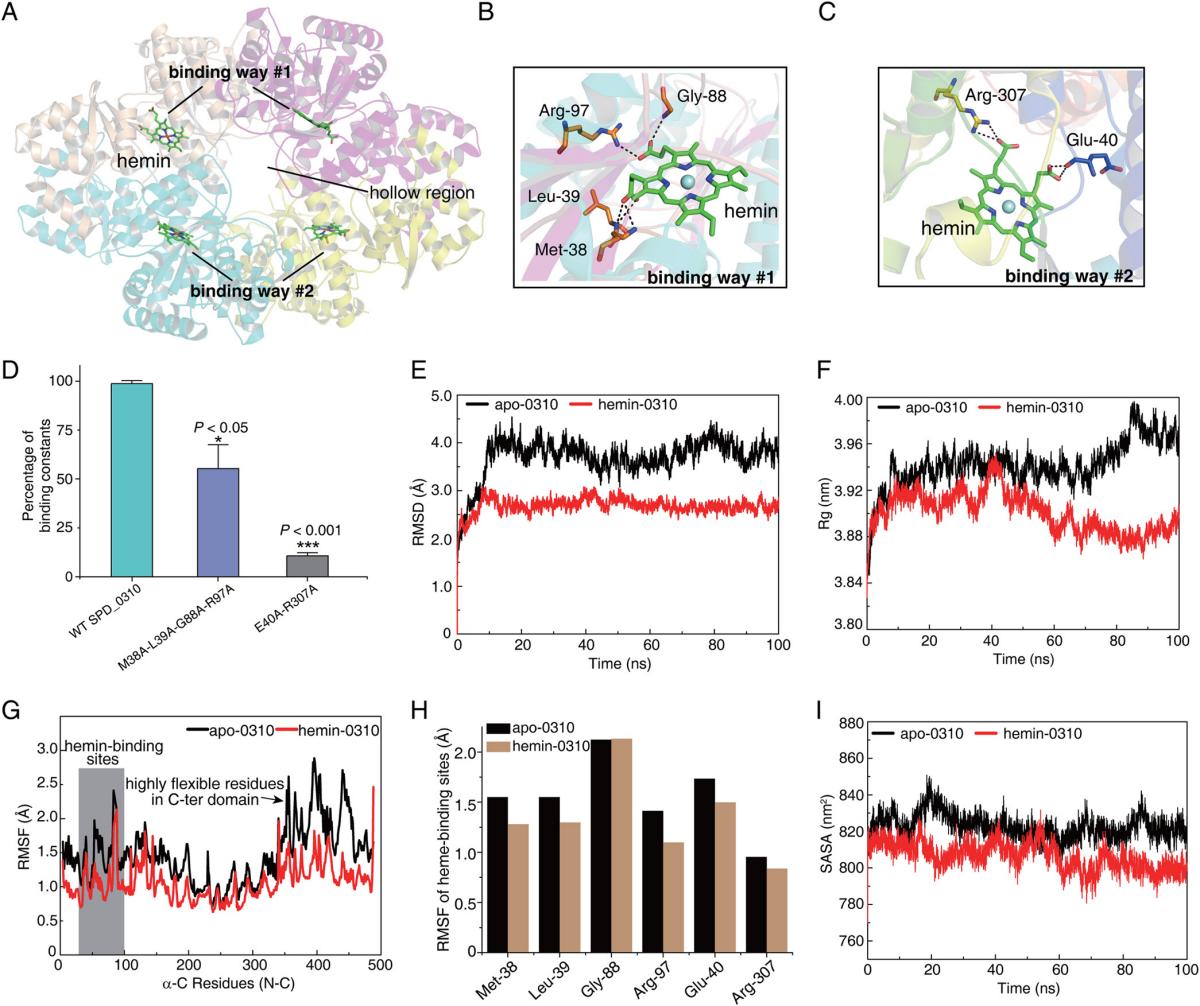
Molecular docking between SPD_0310 and hemin and atomic characterization of structural changes in MD simulation. (A) Global display of docking results between apo-0310 and hemin. (B and C) The details of two hemin binding ways of SPD_0310. The black dotted lines represent hydrogen bonds. (D) The percentage of binding affinity caused by the binding of WT SPD_0310, M38A-L39A-G88A-R97A, and E40A-R307A with hemin. (E) The time evolution of the average RMSD of the C-alpha atoms in the apo-0310 (black) and hemin-0310 (red) simulations. (F) The time evolution of the C-alpha average of the Rg values. (G) The RMSF of C-alpha for all residues calculated over the 100-ns trajectory in the absence and presence of hemin. (H) The comparison of RMSF fluctuation of the residues of apo-0310 and hemin-0310 hemin-binding sites. (I) Time evolution of SASA values of apo-0310 and hemin-0310 in MD systems. The results in panel D were displayed as means ± SD, and the significance of differences was determined by two-tailed Student’s *t* test (*, *P < *0.05; ***, *P < *0.001).

To predict the hemin-binding site of the SPD_0310 protein, we used apo-0310 as a receptor and hemin as a ligand for molecular docking (Fig. S2D). The tetramer crystal structure of SPD_0310 indicated that there was a concave active pocket in the N-terminal domain of the protein; it might be the main hemin-binding region (Fig. 8A). The results showed that the two ways of hemin binding were identified in the N-terminal domain, which might be determined by the spatial configuration of homodimers A and B. The corresponding ligand-binding sites were Met38, Leu39, Gly88, and Arg97 in way 1 (Fig. 8B) and Glu40 and Arg307 in way 2 (Fig. 8C). The reference values of binding free energy of the two binding ways were as low as –5.78 kcal/mol and –5.64 kcal/mol, separately (Fig. S2D), indicating that the results of molecular docking were highly reliable. To verify the two binding ways predicted by molecular docking, we selected the key residues (M38A-L39A-G88A-R97A and E40A-R307A in the two ways) to construct multisite mutant proteins and performed fluorescence titration of hemin. The calculated results of fluorescence quenching showed that the binding constants for hemin of mutants M38A-L39A-G88A-R97A and E40A-R307A were only 56% (*P < *0.05) and 11% (*P < *0.001) of that of WT SPD_0310 (Fig. 8D), respectively. This result indicates that the two predicted binding ways were reasonable.

To evaluate the effect of hemin binding on the conformational changes of the tetramer protein, we performed molecular dynamics (MD) simulations with a total time of 100 ns on apo-0310 and hemin-0310. The results showed that hemin-0310 reached the equilibrium state at 10 ns; in contrast, the root mean square displacement (RMSD) of apo-0310 fluctuated greatly, suggesting its conformational instability in the absence of hemin (Fig. 8E). The radius of gyration (Rg) within the period of equilibrium also suggested a more flexible structure of apo-0310 compared to hemin-0310 (Fig. 8F). Moreover, hemin-0310 showed lower root mean square of fluctuation (RMSF) values than apo-0310 (Fig. 8G), indicating higher rigidity. However, the RMSF value of the C-terminal domain was more varied than that of the N-terminal domain, suggesting that the C-terminal domain has higher flexibility, which is echoed by the high ΔGdiss values of interface A in homodimer A (Table 2). Next, we calculated the RMSF of the hemin-binding residues of two ways for details; the four residues of way 1 (Met38, Leu39, Gly88, and Arg97) and the two residues of way 2 (Glu40 and Arg307) all showed reduced RMSF values after hemin binding (Fig. 8H). The solvent-accessible surface area (SASA) data were derived from the evolution of conformational change over the simulation time (Fig. 8I). The average SASA of apo-0310 (822.39 Å^2^) was significantly higher than that of hemin-0310 (805.71 Å^2^) within the simulation time of 10 to 100 ns (Fig. 8I). A larger SASA value facilitated the interaction between the tetramer and hemin ligand, and the denser structure was beneficial for hemin storage. These results indicate that hemin binding not only stabilizes the N-terminal domain directly, but also regulates the flexibility of the C-terminal domain indirectly through the allosteric effect.

## DISCUSSION

Iron is regarded as an essential trace element in pathogenic bacteria and is involved in the formation of bacterial biofilms and bacterial virulence (32, 33). Since there are very few free iron ions in host cells, bacteria usually compete for heme in heme-binding proteins in host cells to support their own metabolism (34, 35). The typical iron transporters of S. pneumoniae include PiaABC, PiuABC, PitABC (14, 20, 36), etc. Interestingly, in our previous study, we found that the Δ*piaA* Δ*piuA* Δ*pitA* triple mutant strain could survive in normal medium (20), indicating that there are other iron transport systems that compensate for iron. In fact, most bacteria have evolved mechanisms to transport ferric iron (Fe^3+^) via siderophore receptor systems and to compete for heme from heme-containing proteins by infecting host cells (37–40). Bacterial GAPDH can interact with human hemoglobin and plasminogen to compete for hemin-iron and promote infection in the host cells (6, 18). Therefore, survival of the triple mutant strain might stem from hemin being transported to the cytoplasm through GAPDH. However, the fate of hemin after its transport into cells is unknown. The maintenance of iron homeostasis (preventing cytotoxicity caused by excessive iron levels) is an adaptive strategy evident in species of bacteria in which hemin storage proteins, such as HutZ of the bacterium Vibrio cholerae and HmuY of Porphyromonas gingivalis (41, 42), have evolved.

**FIG 9:**
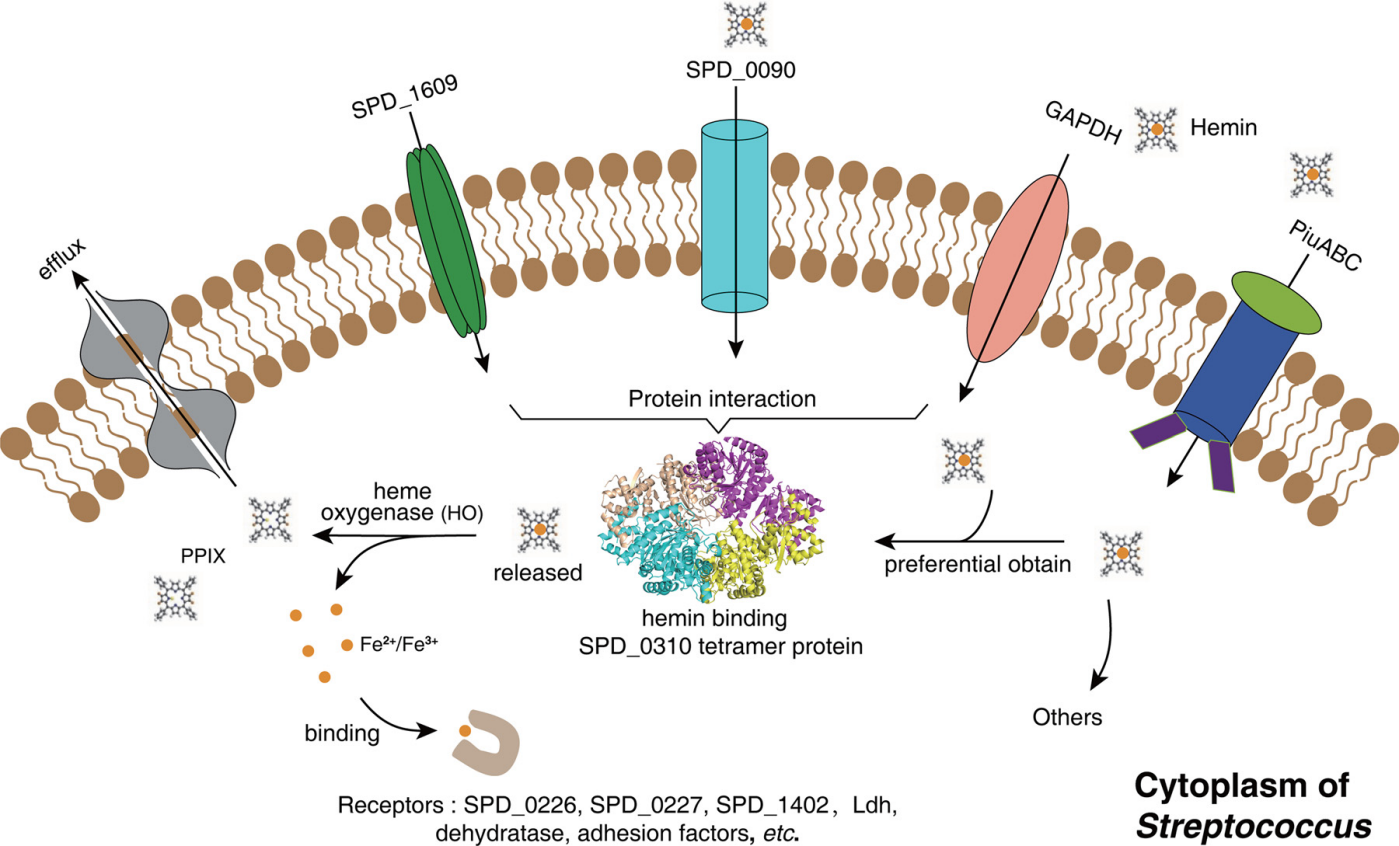
Schematic overview of SPD_0310 function. Hemin transport into cytoplasm depends on GAPDH and PiuABC transporters. Hemin was preferentially obtained by the tetramer SPD_0310 via interaction with GAPDH and SPD_0090. In order to supplement iron ions *in vivo*, the released hemin was catalyzed by heme oxygenase to generate iron ions and PPIX. Subsequently, iron ions were bound to receptor proteins to facilitate their bioactivities, especially virulence-related proteins, and PPIX was excluded because excessive PPIX causes cytotoxicity to cells. Overall, the knockout of the *spd_0310* gene will cause a decrease of intracellular iron content and thus seriously reduce the virulence of S. pneumoniae to host.

SPD_0310 is defined as the UPF0371 family protein in the NCBI protein and UniProt databases. However, the biological functions of this family have not yet been reported. In the current study, we analyzed the universality of UPF0371 family proteins and found that more than 98.3% of homologous proteins were enriched in Gram-positive bacteria. Furthermore, we presented the tetramer structure of SPD_0310 with 2.7 Å, showing the double domain of each monomer and the exact intermolecular forces of the homotetramer. Further, ICP-MS combined with biochemical experiments confirmed that the ratio of tetramer protein to hemin was 1:4. Therefore, considering the binding ratio of receptor to ligand, the tetramer protein has more advantages than the monomer protein in hemin binding. We confirmed by using SPR and UV spectroscopy that hemin is the specific substrate of the SPD_0310 protein. The kinetic detection results of the stopped flow method showed that SPD_0310 binding with hemin was a fast second-order reaction (approximately 0.5 and 7.0 s). Moreover, the results of docking and MD simulations confirmed that hemin may be directly bound to the N-terminal domain in two modes, which can regulate the flexibility of C-terminal residues and stabilize the overall conformation of the protein. Compared with the hemin-bound state, the apo-0310 tetramer may act as a relaxed state in cells. Therefore, we speculated that SPD_0310 may store hemin in S. pneumoniae cells (Fig. 9). We found that cytoplasmic protein SPD_0310 has a special tetramer structure, has strong hemin-binding ability, and interacts with many iron transporters anchored on the membrane, which facilitates hemin transfer from the phospholipid bilayer to the cytoplasm.

How does hemin-saturated SPD_0310 redistribute hemin in cells? Once internalized, hemin would be cleaved and released iron through a redox process (39). In general, hemin is divided into protoporphyrin IX (PPIX) and iron ions by heme oxygenase (HO), followed by uptake by Fe-transporters (34, 43). We also found that SPD_0310 interacts with cytoplasmic iron-binding proteins, such as SPD_0226 and SPD_0227, which may be involved in the redistribution of iron. Thus, we propose that the novel tetramer protein has potential hemin-binding capacity, which is essential for Gram-positive bacteria to maintain iron homeostasis in an iron-deficient environment.

Importantly, we have also shown that Δ*spd_0310* attenuated the ability of adherence or invasion of host cells in mouse infection experiments. The iron concentration in WT-D39 cells was approximately 60 ng/mg, which ensures normal growth, whereas only 40 ng/mg iron in the Δ*spd_0310* strain was detected by ICP-MS (*P < *0.05). This low iron concentration induced by the deletion of *spd_0310* seriously affected iron homeostasis in bacteria and the expression of crucial virulence proteins (for example, *ply*, *cbpA*, *pspA*, and *pcpA*). Moreover, no SPD_0310 homolog protein was found in the human genome. Therefore, our results indicate a novel target for antimicrobial drugs against pathogenic Gram-positive bacteria.

In summary, we found and confirmed a hemin-binding protein, SPD_0310, which affects iron homeostasis and bacterial virulence. The protein has the tetramer crystal structure of SPD_0310, revealing its hemin-binding characteristics through biochemical characterization *in vitro*. Our results demonstrate the possibility of SPD_0310 as a potential antibacterial drug target and provide further direction for investigations of virulence mechanisms in bacteria.

## MATERIALS AND METHODS

### Sequences analysis and evolutionary studies.

The protein sequence of SPD_0310 from S. pneumoniae D39 was used as the seed to query the uniprotrefprot (v.2019_09) database using the HMMER tools (44, 45). The homologous proteins of SPD_0310 were found in different organisms with high bit scores and more than 35% sequence identity. The evolutionary history was inferred by using the maximum likelihood method based on the Jones-Taylor-Thornton (JTT) matrix-based model. We selected only 69 representative amino acid sequences to construct the phylogenetic tree in MEGA7 (46). The initial tree for the heuristic search was obtained automatically by applying neighbor-joining and BioNJ algorithms to a matrix of pairwise distances estimated using a JTT model and then selecting the topology with the superior log likelihood value. The tree is drawn to scale, with branch lengths measured in the number of substitutions per site. Then, a multiple sequence alignment was performed using partial proteins with high scores through the software package Clustal-X 2.0.

### Crystallization, data collection, and structure determination.

Crystallization of SPD_0310 was performed using the hanging drop vapor diffusion method by mixing 1 μL protein solution (about 15 mg/mL) and 1 μL reservoir solution at 16°C. Crystals of SPD_0310 were grown from drops consisting of a reservoir solution of 2.0 M ammonium sulfate and 0.1 M HEPES (pH 7.5) in about 24 h. A 2.7-Å resolution diffraction data set was collected at –175°C at biological small-angle X-Ray scattering (BioSAXS) BL19U1 of the Shanghai Synchrotron Radiation Facility (SSRF), China, and was processed, integrated, and scaled together with HKL3000 (47). The statistics of the diffraction data are summarized in Table 1. Initial phases were obtained by the molecular replacement (MR) method implemented in Phenix (48) using protein DIP2346 (PDB code 3BH1) as the search model, and the initial model was built manually using coot (49). Structure refinement was carried out using Phenix and REFMAC5 (48, 50). The stereochemistry of the structure model was analyzed using MolProbity (51). Structure analysis was carried out using programs in CCP4 (52). The structure figures were prepared using PymoL-1.7 (http://www.pymol.org). The statistics of the structure refinement and the quality of the structure model are also summarized in Table 1.

### Construction, expression, and purification of SPD_0310.

To construct a recombinant expression plasmid, the restriction enzyme cutting sites of BamH I/Sal I were introduced to the PCR primers (Table S1). The *spd_0310* gene was cloned from the S. pneumoniae D39 genomic DNA. The confirmed PCR products of *spd_0310* were digested with BamH I/Sal I and ligated into the pGEX-4T-1 plasmid (Invitrogen). The positive recombinant plasmid pGEX-4T-1-*spd_0310* was confirmed by DNA sequencing (Invitrogen) to ensure no mutation and then transformed into Escherichia coli BL21(DE3) expression cells. Next, the final concentration of 0.5 mM IPTG (isopropyl-β-d-thiogalactopyranoside) was added to LB medium (500 mL) when the optical density at 600 nm (OD_600_) reached 0.6 to 0.7. Bacteria were harvested by centrifugation (6,000 × *g*, 10 min) and lysed by sonication, and the supernatant was collected by centrifugation (10,000 × *g*, 30 min). A GST affinity column was applied to separate the fusion protein SPD_0310-GST from E. coli total proteins, and then the GST tag was removed with thrombin (Sigma-Aldrich). Purified SPD_0310 protein was detected by 12% SDS-PAGE and identified with mass spectroscopy (ABI 4800 MALDI-TOF/TOF) (Fig. S1).

10.1128/msystems.00221-22.3 TABLE S1 The primers were used for construction of mutant strains and WT and mutant proteins. Download Table S1, DOCX file, 0.01 MB. Copyright © 2022 Cao et al. 2022 Cao et al. https://creativecommons.org/licenses/by/4.0/ This content is distributed under the terms of the Creative Commons Attribution 4.0 International license.

### DLS measurements.

The DLS method based on a Zetasizer Nano ZS device (Malvern Instruments Ltd., UK) was performed to measure the particle size of SPD_0310 under different states (53). The protein particles in the solution buffer (1× phosphate-buffered saline [PBS]) continuously undergo Brownian motion, and the light scattering intensity values of the protein samples is transformed into particle size distribution (54, 55). The 1× PBS buffer was prepared with ultrapure water, and impurities were filtered using 0.22-μm filtration. Fresh SPD_0310 in the native state was diluted to the final concentration of 3 μM with 1× PBS. Moreover, 10% SDS was used to interrupt native SPD_0310 aggregation to form the monomer protein. Then, we detected the size distribution of the protein sample in the solution with the following parameters: 25°C, 294.5 derived count rate, rely on distribution only, water solvent, 0.894 intercept, 50-s duration.

### Surface plasmon resonance (SPR).

The SPR experiments were performed using an OpenSPR instrument (Nicoya Lifesciences, Canada) to determine the interactions between SPD_0310 and analyte molecules, such as BSA protein (negative control), bovine lactoferrin (positive control), hemin, GAPDH, SPD_1609, SPD_0090, SPD_0226, and SPD_0227 (56, 57). First, the carboxyl end of SPD_0310 (100 μg/μL, 300 μL) was immobilized on a gold nanoparticle COOH sensor chip, using 1× PBS buffer as a mobile phase to rinse the SPD_0310 that was not integrated on the chip. The constant flow rate was always controlled as 30 μL/min. Next, the analyzed molecules were diluted with 1× PBS to different concentration gradients (volume, >200 μL). Each sample that interacted with the target SPD_0310 protein was removed with 0.5 to ∼10 mM HCl. The combination of each sample and SPD_0310 on the chip generated different curves according to the concentration. The dissociation constant (*K_D_*) was calculated after fitting these curves using a simple 1:1 model (A+B ↔ AB).

### Hemin-agarose-binding assay.

The magnetic beads of hemin-agarose or apo-agarose (Sepharose 4B, Sigma-Aldrich) were washed using 1 mL solution buffer (100 mM Na/K phosphate and 200 mM NaCl, pH ∼6.0) and centrifuged at 800 × *g* for 5 min. Then, 4 μg SPD_0310 protein was incubated with hemin-agarose (experiment group) or apo-agarose (negative control) beads for 2 h at 37°C. During the incubation process, it is necessary to slightly sway the mixture to ensure adequate combination, followed by slight centrifugation to separate the sediments from the supernatants. The precipitation is the bound sample, and the supernatant is the flow through (FT) sample. In addition, 0.2 μg purified SPD_0310 was used as a positive control (input sample) (58). Next, SDS-PAGE analysis was used to determine whether the protein was combined with hemin.

### UV spectrum.

A UV spectrophotometer (Evolution 300, Thermo) was used to detect the binding of hemin to protein. The specific absorption peaks of the apo form protein and hemin are shown at 280 nm and 400 nm, respectively. However, the specific absorption peak of hemin-binding protein is located at 410 nm of the visible region. Thus, the peak shift of 10 nm is a crucial indicator to determine whether protein can bind hemin ligand. The preparation of the experiment sample was a follows: 50 μM SPD_0310 was incubated with 100 μM hemin for 6 h and dialyzed and centrifuged (10,000 × *g*, 20 min) to remove excess hemin ligands. Hemin-0310 with a final concentration of 20 μM was used for UV analysis. Also, samples of 20 μM apo-0310 and 20 μM hemin were prepared using the same buffer (20 mM Tris-HCl and 100 mM NaCl, pH 7.4). The UV spectra were scanned from 200 to 600 nm, and each experiment was repeated three times.

### Circular dichroism (CD) spectroscopy.

To determine the secondary structures of apo-0310 and hemin-0310, we collected the circular dichroism data from 260 to 190 nm at 25°C using a CD spectrometer (Chirascan, Applied Photophysics Ltd.). The protein samples were dissolved in 20 mM Tris-HCl, pH 7.4. The CD spectrum of each protein was scanned three times and the average value was obtained. The calculation of the secondary structure content was based on CDPro software. Detailed parameters were as previously described (29, 36).

### ICP-MS analysis.

The WT-D39, Δ*spd_0310*, and WT-p169-*spd_0310* strains were cultured in 10 mL normal THYE medium. All bacteria were collected at an OD of ∼0.3 (5,000 × *g*, 10 min). Bacterial precipitation was washed three times with chelated 1× PBS buffer to facilitate the elimination of metal ions in the medium. The bacterial cells were freeze-dried with a ScanVac freeze dryer, and then the dry weight of the cells was weighed; 35% HNO_3_ (1.5 mL) was used to dissolve the dry bacterial precipitation. To remove the HNO_3_ in solution as much as possible, the samples were boiled for 10 min at 100°C. Next, the samples were centrifuged for 30 min (13,000 × *g*), and the metal contents were determined by ICP-MS. Three biological replicates were performed for each sample. The normalization analysis of the data was based on the dry weight of the cells.

### Stopped-flow absorbance kinetics.

The hemin-binding kinetics of SPD_0310 were measured by a fluorescence mode of stopped-flow reaction analyzer (Chirascan SF.3, UK), adding 20 μM apo-0310 (500 μL) protein sample into injector A and 20 μM hemin (500 μL) ligand sample into injector B. The instrument parameters were set as follows: the excitation wavelength was 280 nm, the detection wavelength was 330 nm (320-nm high-flux filter), the bandwidth was 1 nm, the scanning time was 60 s, the fluorescence intensity was <8.0, and the voltage was <800 V; repeated 10 times for each scan. To get accurate data analysis, the scanning background caused by the buffer (20 mM Tris-HCl and100 mM NaCl, pH 7.4) was subtracted. The collected data were analyzed using a second-order exponential equation, *y* = *y*_0_ + A_1_ × exp(–*x*/*t*_1_) + A_2_ × exp(–*x*/*t*_2_) to obtain the apparent rate constants.

### Construction of the *spd_0310* knockout or complement strain.

Homologous recombination is a mature strategy for constructing gene knockout strains. The target *spd_0310* gene was replaced with the erythromycin (*erm*) gene in S. pneumoniae D39. The primer sequences of P1, P2, P3, and P4 were designed to obtain long flanking homologous (LFH) segments containing the *erm* gene and are listed in Table S1. We transformed the LFH products obtained by PCR into WT-D39 competent cells. Next, positive clones were screened using a Columbia blood plate with 0.25 μg/L Erm.

Shuttle plasmid p169 was a simple RCR plasmid containing chloramphenicol resistance, with a total of 4,436 bases (27). It has been used often for overexpression of target genes in S. pneumoniae because it contains the *veg* promoter, which is a homologous promoter isolated from Gram-positive bacteria (59). In this study, we first introduced the single enzyme cutting site of EcoR I into the PCR primer of the *spd_0310* gene to obtain the target gene fragments. To construct a *spd_0310* complement strain, the p169-*spd_0310* recombinant plasmids were transformed into the *Δspd_0310* strain. The positive clones were screened with a 4-μg/L chloramphenicol-resistant blood plate. The detailed gene knockout and complement procedures were the same as those of our previous studies (14, 22, 29).

### Detection of virulence of WT-D39 and the Δ*spd_0310* strain on a mouse model.

The strains WT-D39, Δ*spd_0310*, and *spd_0310* complement were expanded to 10 mL THYE medium. The bacterial sediments were collected and resuspended with 5 mL 1× PBS to ensure that the OD value was about 0.3, which was equivalent to maintaining the concentration of bacteria at about 1 × 10^8^ CFU/mL (60, 61). Then, 32 female BALB/c mice (4 weeks old) were used and divided into four groups, corresponding to 0.6% NaCl (negative control) and the different bacterial infections mentioned above. Next, 20-μL volumes of WT-D39, Δ*spd_0310*, and *spd_0310* complement strain samples with a final concentration of 1 × 10^8^ CFU/mL were injected into the mice through the tail vein, and the number of bacteria injected was equivalent to about 2 × 10^6^ CFU. The mice were given normal nutritional supplements under the same survival conditions, and the mouse survival proportions were monitored over a 7-day period (62–64). To avoid cross-infection of bacteria, mice infected with the same bacteria were placed in the same box. It was necessary to ensure adequate food and aseptic water. To calculate the survival rate of infected mice, we observed the growth status of mice and recorded the time of their death (65). BALB/c nude mice were cared for under standard conditions according to institutional guidelines. All animal experiments were approved by the Ethics Committee for Animal Experiments of Jinan University.

### Identification of the interacting proteins of SPD_0310 using GST pulldown and MS.

The candidate proteins interacting with SPD_0310 were identified with mass spectrometry (Triple-TOF 5600; AB SCIEX, USA). The GST-gel beads (Sigma-Aldrich) were combined with SPD_0310-GST fusion protein or GST and then incubated with the whole proteins of S. pneumoniae D39 for 10 h at 4°C. Next, the protein-gel beads (mixture) were centrifuged at 2,000 × *g* for 3 min to remove the supernatant. Then, the mixture was washed with 1× PBS three times, incubated with SDS-PAGE loading buffer, and boiled for 10 min and detected by 12% SDS-PAGE gel (66). The protein samples of different lanes were digested in-gel and desalted using desalting column (Bio-Rad, Shanghai, China) and the Acclaim PepMap 100 C_18_ column of the high-pressure liquid chromatography (HPLC) system (Thermo), respectively. After lyophilization, the samples were redissolved using 0.1% (vol:vol) formic acid. Detailed procedures were performed in accordance with our previous study (14). The candidate proteins interacting with SPD_0310 were identified by mass spectrometer. The identified peptides were performed to search against the NCBInr database of S. pneumoniae D39 using Mascot (v.2.2.04; Matrix Science, Boston, MA, USA) (67–69). The specific parameters were as follows: (i) enzyme, trypsin; (ii) fixed modification carbamidomethyl; (iii) unique peptides, ≥2; (iv) peptide length, ≥8 amino acids (aa); (v) variable modification oxidation; (vi) contaminants, trypsin and keratins. Next, the identified candidate proteins were expressed and purified using the GST system to confirm their interaction with SPD_0310. The mass spectrometry proteomics data have been deposited in the ProteomeXchange Consortium via the PRIDE (70) partner repository with the data set identifier PXD030298 and can be accessed with the reviewer account (website, http://www.ebi.ac.uk/pride; username, reviewer_pxd030298@ebi.ac.uk; password, kZePlTsU).

### Real-time quantitative PCR (qRT-PCR).

We collected bacterial precipitates of WT-D39 and the Δ*spd_0310* mutant strain at the logarithmic phase (OD, ∼0.3), and total RNA was extracted from each strain with TRIzol reagent (Invitrogen, USA). The RNA concentration was determined using a NanoDrop 2000 UV-VIS spectrophotometer (Thermo Scientific, USA), and the purity was detected by agarose gel electrophoresis. Next, 1 μg of RNA was reverse transcribed to produce cDNA in strict accordance with the protocol of the reverse transcription kit (cDNA synthesis SuperMix kit; TransGen Biotech, China) (30); sterile water without RNase and ribozyme was used in this process. We used 16S rRNA as an internal reference because its expression was not affected by *spd_0310* gene knockout in our preexperiment. Then, qRT-PCR was carried out by using EvaGreen dye (Bio-Rad, USA) in a MiniOpticon real-time PCR system (Bio-Rad, USA). The threshold cycle (*C_T_*) values of 16S rRNA, *ply*, *cbpA*, *pspA*, and *pcpA* were recorded, and relative quantification of target genes was performed according to the 2(–Delta Delta C[T]) method (71). The primer sequences are shown in Table S1. All data were evaluated with three independent biological experiments.

### MD simulation.

To predict the active site of SPD_0310 for hemin binding, flexible docking between receptor SPD_0310 and hemin was performed using LeDock software (http://www.lephar.com/download.htm) and then searched for the lowest energy-binding conformation between ligand and receptor (72, 73). In this study, the MD simulations for the tetramer of apo-0310 and hemin-0310 were carried out using the GROMACS 2020.4 package at a constant temperature of 300 K. Based on the charmm27 molecular force field and Simple Point Charge (SPC) water model, the apo-0310 and hemin-0310 systems were performed for a total time of 100 ns, respectively. First, the crystal structure of SPD_0310 was placed in the cubic periodic box as the initial conformation, and the minimum distance between the protein and the box boundary was set at 1.2 nm. The periodic boundary conditions of the simulation system were suitable for X/Y/Z directions. We added 0.15 mol/L NaCl salt solute to the solvent (SOL) to neutralize the charge of the protein system. We used the particle mesh Ewald (PME) method to compute electrostatic interactions and the leap-frog algorithm to calculate the atomic motion of each atom (29). The steepest descent energy method was used to perform the 400-step energy minimization, and then the conjugate gradient method was used to execute 25,000-step energy minimization. We performed 50-ps position constraint simulation for each MD system and set a random initial speed (74).

### Data and material availability.

The atomic coordinates and structure factors of the SPD_0310 structure have been deposited in the Protein Data Bank with accession code 7F00. All data are available in the main text or the supplemental materials.
