# Supplementary material for: Identification and Tetramer Structure of Hemin-Binding Protein SPD_0310 Linked to Iron Homeostasis and Virulence of Streptococcus pneumoniae
Source: mSystems. 2022 Apr 13;7(3):e00221-22. doi: 10.1128/msystems.00221-22 (PMC9238395; doi:10.1128/msystems.00221-22)
Supplement: TABLE S1 [file msystems.00221-22-st001.docx]

**Table S1. The primers were used for construction of mutant strains and WT and mutant proteins.**

| Primer | Sequence (5′-3′) | | Comments |
| --- | --- | --- | --- |
| 0310-P1 | AAGAGGCTGTGGTCCTTGTAG | | gene knockout |
| 0310-P2 | TTTATCTCACCAGTCTTTCCAC | | gene knockout |
| 0310-P3 | ATCAAACAAATTTTGGGCCCGGAGATTTCAACCTCTCCAGCCT | | gene knockout |
| 0310-P4 | ATTCTATGAGTCGCTGCCGACTACCTCTGACCTCTATTATAATA | | gene knockout |
| Erm-F | AGTCGGCAGCGACTCATAGAAT | | gene knockout |
| Erm-R | CCGGGCCCAAAATTTGTTTGAT | | gene knockout |
| pIB169-spd0310-F | GAGACCGCGGTCCCGAATTCATGAAAAAACAAGCTTTTAGTTC | | overexpression |
| pIB169-spd0310-R | CGGGTACCGAGCTCGAATTCTTAGTGATGGTGATGGTGATGCTTACGA TATAAGCGGTCGTAT | | overexpression |
| 0310-F | CGTATCGGATCCATGAAAAAACAAGCT | protein purification | |
| 0310-R | GCGGCGGTCGACTTACTTACGATATAA | protein purification | |
| M38A-L39A-F | GGTAAAGCGGCAGAAGATTTCCACGCTGCTCG | | mutant |
| M38A-L39A-R | ATCTTCTGCCGCTTTACCGCCAAACTCTAAGT | | mutant |
| G88A-R97A-F | ACTTAGCCATTTCTTATGACCAAGAAG | | mutant |
| G88A-R97A-R | GTCAATCAAAGCAAGAACTTCTTGGTCATAAG | | mutant |
| E40A-F | TGTTAGCAGATTTCCACGCTGCTCGTGTCCTTC | | mutant |
| E40A-R | AAATCTGCTAACATTTTACCGCCAAACTCTAAG | | mutant |
| R307A-F | ATCCGCGCTTACTATCAAACAGTTCTTGACTTC | | mutant |
| R307A-R | TAGTAAGCGCGGATGATTTCTTGTTTAGAAGC | | mutant |
| ply-R | ATCGCTACTTGCCAAACCAG | | qRT-PCR |
| ply-F | CTACCAACGACAGTCGCCTC | | qRT-PCR |
| cbpA-R | AGGCGACATTTTGGGTATGTT | | qRT-PCR |
| cbpA-F | TTGCCAGTCTTGTTATGGGAA | | qRT-PCR |
| pspA-R | GAGCAGCTTTTGCATCATCT | | qRT-PCR |
| pspA-F | ATCTCCCGTAGCCAGTCAGT | | qRT-PCR |
| pcpA-R | CCTCAGTTGATGGTGTTTTGTTT | | qRT-PCR |
| pcpA-F | TCTTTTGTCTCCTTAGGCGTTTT | | qRT-PCR |
| 16S rRNA-F | CTGCGTTGTATTAGCTAGTTGGTG | | qRT-PCR |
| 16S rRNA-R | TCCGTCCATTGCCGAAGATTC | | qRT-PCR |
